# What affects the magnitude of age-related dual-task costs in working memory? The role of stimulus domain and access to semantic representations

**DOI:** 10.1177/1747021820970744

**Published:** 2020-11-27

**Authors:** Agnieszka J Jaroslawska, Stephen Rhodes, Clément Belletier, Jason M Doherty, Nelson Cowan, Moshe Neveh-Benjamin, Pierre Barrouillet, Valerie Camos, Robert H Logie

**Affiliations:** 1 School of Philosophy, Psychology and Language Sciences, University of Edinburgh, Edinburgh, UK; 2 School of Psychology, Queen’s University Belfast, Belfast, UK; 3 Department of Psychological Sciences, University of Missouri, Columbia, MO, USA; 4 Faculty of Psychology and Educational Sciences, University of Geneva, Geneva, Switzerland; 5 Department of Psychology, University of Fribourg, Fribourg, Switzerland

**Keywords:** Working memory, dual-task, ageing, storage, processing

## Abstract

Although there is evidence that the effect of including a concurrent processing demand on the storage of information in working memory is disproportionately larger for older than younger adults, not all studies show this age-related impairment, and the critical factors responsible for any such impairment remain elusive. Here we assess whether domain overlap between storage and processing activities, and access to semantic representations, are important determinants of performance in a sample of younger and older adults (*N* = 119). We developed four versions of a processing task by manipulating the type of stimuli involved (either verbal or non-verbal) and the decision that participants had to make about the stimuli presented on the screen. Participants either had to perform a spatial judgement, in deciding whether the verbal or non-verbal item was presented above or below the centre of the screen, or a semantic judgement, in deciding whether the stimulus refers to something living or not living. The memory task was serial-ordered recall of visually presented letters. The study revealed a large increase in age-related memory differences when concurrent processing was required. These differences were smaller when storage and processing activities both used verbal materials. Dual-task effects on processing were also disproportionate for older adults. Age differences in processing performance appeared larger for tasks requiring spatial decisions rather than semantic decisions. We discuss these findings in relation to three competing frameworks of working memory and the extant literature on cognitive ageing.

Working memory can be characterised as a system for the simultaneous storage and processing of information (e.g., Baddeley, 2012; Barrouillet & Camos, 2015; Cowan, 1999; Logie, 2011). On this account, working memory has the dual function of maintaining information that is no longer accessible in the environment (which we refer to as memory) and performing other cognitive activities which require the transformation of information (which we refer to as processing), as opposed to merely storing the information given. A persistent question in the working memory literature has been whether older adults show poorer ability than younger adults to balance these two functions of memory and processing when both are required (e.g., Baddeley et al., 1986; Gick et al., 1988; Logie et al., 2004; Rhodes et al., 2019; Riby et al., 2004; Verhaeghen et al., 2003; Wright, 1981).

Working memory tasks typically require dual-tasking in which individuals hold some information in mind (the “memory” component) while also undertaking some other task (the “processing” component). Most of the evidence gathered using such tasks points to the presence of an effect of processing on older adults’ working memory performance that is disproportionally large compared with young adults (for a review see Jaroslawska & Rhodes, 2019), often referred to as an age-related dual-task cost. Nevertheless, there remains substantial, and largely unexplained, variability in the magnitude of the reported age effects with some studies showing no differential effect of age on dual-task performance (e.g., Baddeley et al., 1986; Kaschel et al., 2009; Logie et al., 2004). Somberg and Salthouse (1982) showed that age-related dual-task costs could be attributed to age differences in performance on single-task performance. They recommended that the demand of each single task is adjusted (titrated) to the ability of each participant. Any drop in performance when the two tasks are performed together could then be attributed specifically to a dual-task cost to performance, not to an artefact of group or individual differences in single task performance. In a recent meta-analysis, Jaroslawska and Rhodes (2019) showed that age-related increases in dual-task costs were mitigated when the demands of each task were tailored (i.e., titrated) according to the ability of each participant, as recommended by Somberg and Salthouse (1982). However, other reports that have included titration of both storage and processing have found clear evidence of age-related differences (e.g., Bier et al., 2017; Rhodes et al., 2019). Therefore, other possible critical factors responsible for the disproportionate age effect in dual-task costs remain subject to debate. To address this long-standing controversy, we considered whether the nature of the tasks that are combined might be crucial. To do so, we investigated the impact on age-related dual-task costs of domain overlap (verbal/non-verbal) between storage and processing activities, and access to semantic representations, in a sample of younger and older adults.

Here, we focus on two aspects of study methodology that the literature suggests may lead to a systematic modulation of the size or presence of age differences. Specifically, we assessed whether the extent to which processing tasks that are concurrent with memory tasks disrupt memory performance with age is driven by the type of mental operations required to perform the task (i.e., spatial decisions or semantic decisions) and the type of stimuli used for both activities (i.e., verbal or non-verbal). Note that, although not addressed directly in this study, there are other potential moderators of age differences in managing simultaneous storage and processing demands. One such factor is the trade-off between speed and accuracy which often differs as a function of age, with older adults sacrificing speed for accuracy (e.g., Baron & Mattila, 1989; Hertzog et al., 1993; Salthouse, 1979). We mitigated the impact of age differences in speed/accuracy trade-offs by titrating single task demands for each participant. This issue is covered directly in a corresponding set of experiments by Rhodes et al. (under review) that examined the impact of trial timing manipulations on age differences in managing simultaneous storage and processing demands in a working memory task. In summary, the results of those experiments suggested that the time available for each trial did not account for age differences in dual-task performance.

The aims of this work are twofold. The first aim is to establish whether age-related dual-task costs can be attenuated or amplified by varying the nature of the processing activity. The second is to evaluate our findings in light of three competing theoretical frameworks of working memory that make conflicting claims about how concurrent storage and processing is achieved (for review see Cowan, 2017; Cowan et al., 2020; Logie et al., in press-b). Some theorists claim that working memory is partitioned into several code- or modality-specific resources, which are distinct from domain-general executive resources that facilitate the processing of information (e.g., Baddeley, 2012; Baddeley & Logie, 1999; Logie, 2011; Vandierendonck, 2016), or indeed that executive functions arise from the interaction between domain-specific modules (Eisenreich et al., 2017; Hazy et al., 2006, 2007; Logie, 2016). Other researchers propose that performance is supported, in part, by a common attentional resource that can be shared between competing demands and across stimulus domains (e.g., Barrouillet et al., 2004; Barrouillet & Camos, 2015; Cowan, 1988, 2010). We consider whether these existing frameworks of working memory can adequately handle the pattern of dual-task interference observed in this study and discuss how this evidence could be used to reconsider theoretical assumptions about storage and processing in working memory.

## Possible moderators of age differences in the effects of processing on storage in working memory

### Stimulus domain

Stimulus domain is an important determinant of age-related differences in performance on working memory tasks. Jaroslawska and Rhodes (2019) found that verbal tasks produced smaller age effects than the corresponding tasks using visuospatial materials (see also Hale & Myerson, 1996; Jenkins et al., 1999, 2000; Myerson et al., 1999). This result is in line with the robust dissociation between verbal and non-verbal age effects: verbal skills appear to be relatively spared by ageing, whereas non-verbal skills typically exhibit steeper age-related declines (e.g., Hale & Myerson, 1996; Jenkins et al., 1999, 2010; Myerson et al., 2003; Park et al., 2002). Furthermore, in most studies that contrast modality, within-modality dual-tasking tends to result in larger concurrence costs than between-modalities dual-tasking (e.g., Pashler & Sutherland, 1998). For example, the maintenance of verbal material is disrupted to a larger extent by a processing task requiring verbal operations compared with when the processing task involves visuospatial operations (e.g., Bayliss et al., 2003; Cocchini et al., 2002; Farmer et al., 1986; Logie, 1986; Logie et al., 1990; Meiser & Klauer, 1999; Thalmann & Oberauer, 2017; Vergauwe et al., 2010).

However, it is not clear that age differences in concurrent storage and processing performance are exacerbated when the two tasks overlap in domain. For example, when Jenkins et al. (2000) combined tasks that were assumed to involve verbal or spatial memory with tasks that were assumed to involve either verbal or spatial processing, they found no evidence that domain-specific interference was exacerbated in older adults. Jaroslawska and Rhodes (2019) found some evidence that overlap, particularly when both tasks used non-verbal stimuli, was disproportionally detrimental to older adults’ dual-task performance. However, they also observed that studies with domain overlap tended to be those that did not titrate the level of single-task demand ahead of combining the tasks. Once this was accounted for in a meta-regression, the role of domain overlap in age differences in dual-task costs was no longer significant. These findings hint at an overall role of stimulus domain in age differences but leave the question of its role in modulating the extent of costs to concurrent storage and processing open to debate (see also Hale & Myerson, 1996; Jenkins et al.,1999, 2000; Logie et al., 2015; Myerson et al., 1999).

### Impact of access to semantic representations

Indices of semantic long-term memory-based retrieval, such as vocabulary, increase as people grow older and remain relatively stable into old age (e.g., Grady, 2012; Nyberg et al., 1996; Park et al., 2002; Rönnlund et al., 2005; Singer et al., 2003). A meta-analysis of 210 studies indicated that older adults score substantially higher on vocabulary tests than younger adults (Verhaeghen, 2003). This effect is typically explained in terms of older adults continuing to expand their semantic knowledge throughout their lives.

In the broader literature on ageing and dual-tasking, studies using the psychological refractory period (PRP) effect have shown that there are certain task responses that may remain almost unaffected by a concurrent cognitive demand across adult ageing. For example, Lien et al. (2006) found evidence of superior dual-task processing for older versus younger adults on tasks with which older adults have a lot of experience (e.g., word recognition), but that benefit did not extend to a relatively novel activity (i.e., a box-width judgement). These findings are in line with the idea that the efficiency of lexical access improves with age and that older adults can carry out word recognition without compromising the performance of an ongoing processing task. Moreover, Fisk and Sharp (2004) reported the absence of a decline with age in the efficiency of access to long-term memory. Rhodes et al. (2019) combined a serial recall task with arithmetic processing during the retention interval and adjusted the difficulty of each task to the individual ability of each participant. Of interest here is the asymmetrical nature of the concurrence cost which appeared strongly for the memory task and was not at all clear for the processing task. Rhodes et al. (2019) speculated that this effect may have been driven by the possibility that arithmetic verifications could have been performed by direct retrieval of well-learned arithmetic solutions stored in semantic memory. A preliminary analysis of participant reports of strategy use in a related study of younger adults (Doherty et al., 2019) suggested that many participants did indeed use a retrieval strategy for arithmetic, particularly when also trying to retain a verbal memory preload (Belletier et al., under review). In the current experiment we varied the degree to which the processing task draws on semantic memory for generating the correct answer, which may alleviate its demands particularly for older adults and allow them to focus on maintenance activities. This manipulation also allowed us to explore whether healthy older adults show a reduced dual-task cost when combining a memory preload with retrieval of information consolidated in long-term memory over their lifetime, rather than combining memory retention with a less familiar visuospatial task that cannot readily be supported by long-term memory.

## The current study

The work presented here sought to establish whether domain overlap between storage and processing activities, and access to semantic representations, impact on working memory performance in a sample of younger and older adults. We examined whether the serial recall of verbal information varies as a function of the processing task domain and requirements, and as a function of age. To this end, we created four versions of a processing task by controlling the type of stimuli shown and whether or not task performance was contingent upon access to long-term memory representations.

Participants were asked to simultaneously store arbitrary sequences of consonants in mind (memory task) while performing one of four processing activities. Two processing tasks required a semantic decision (*Is it alive or manufactured?*) and two required a spatial decision (*Is it located above or below the centre of the screen?*). The former task required retrieval of semantic information from long-term memory. For the semantic and spatial tasks there was a version with non-verbal stimuli (drawings of animals/objects) and a version with verbal stimuli (names of animals/objects). The memoranda used in all task conditions were verbal (i.e., sequences of consonants). Half of the participants carried out same-domain (i.e., verbal) processing, whereas the other half were instructed to carry out different-domain (i.e., non-verbal) processing. Although age-related effects in dealing with a concurrent task load have been investigated in the past (e.g., Clapp et al., 2011), this is the first study to test the premises about domain overlap and access to semantic representations with task demands titrated to the ability of each participant. By titrating the processing and storage demands to the individual abilities of each participant, we can be more certain that any disproportionate dual-task costs in older adults are not accounted for by age differences in performing each task on its own.

This work resulted from an adversarial collaboration—a cooperative research effort undertaken by three groups of investigators who hold different theoretical views regarding working memory (see Cowan et al., 2020, for an account of this approach, and further details at http://womaac.psy.ed.ac.uk). One of our two key objectives was to evaluate the impact of stimulus domain and reliance on access to semantic memory representations on the magnitude of age-related dual-task costs in light of three competing theoretical frameworks of working memory that make conflicting claims about how concurrent storage and processing is achieved. The three frameworks under discussion are a version of the multiple-components framework (Logie, 2011, 2016), embedded processes (Cowan, 1988, 2005, 2010, 2016), and time-based resource sharing (TBRS; Barrouillet et al., 2004, 2007; Barrouillet & Camos, 2015) models of working memory. The adversarial collaboration process involves reaching consensus on an optimal research design and the corresponding testable, contrasting hypotheses. To facilitate the interpretation of the results, all parties were required to formulate and document their expectations about the outcome of the study prior to data collection. Next, we describe the different predictions from proponents of the three frameworks of working memory.^1^

### Multiple-component model expectations

Baddeley and Hitch (1974), and subsequently Baddeley and Logie (1999), and Logie (2011), proposed that a range of specialised cognitive resources support online cognition. These resources are assumed to include a verbal short-term memory system capable of storing and rehearsing phonological codes, and a visual short-term memory system for visual and spatial representations. Originally, a central executive was proposed as a domain-general processing and control mechanism that coordinated the functioning of the short-term memory systems (Baddeley, 1986). Subsequently, a number of separate executive functions were proposed, including inhibition, updating, task switching, and dual-tasking (Miyake et al., 2000) and these have been suggested to be emergent properties of how different cognitive systems interact and are deployed to meet task requirements (see Logie, 2016; Logie et al., in press-a). Thus, according to the multiple-component account, storage and processing can, under certain circumstances, run in parallel, with little-to-no conflict between the demands. However, one key requirement for minimising the conflict between the two functions is that processing and storage do not both involve materials from the same domain (e.g., both verbal or both visual). A second key requirement is that the capacity of the individual components of working memory should not be exceeded.

According to the multiple-component view of working memory outlined by Logie (2011, 2016; Logie et al., in press-a), serial-ordered recall for visually presented letters at span reflects the use of phonological codes and a phonological rehearsal process. Memory is assumed to be supplemented by a passive visual code for visually presented letters and activated representations of the letters stored in long-term memory (Logie et al., 2016). Both semantic processing tasks are presumed to involve representations activated from long-term memory and some capacity of the visual store. Semantic processing of visually presented verbal items is additionally expected to place a small load on the passive phonological store. Spatial judgements are expected to use capacity of the visual store, but not the phonological loop. In both spatial processing tasks, it is expected that there will be some activation of long-term memory because the verbal and non-verbal visually presented items are meaningful, even if semantic processing of them is not required. As noted earlier, older participants have poorer visuospatial ability than younger participants, but have much less age-related decline in verbal abilities, and access to semantic knowledge may be better as a result of life time experience than for younger participants.

On the basis of these assumptions, there was the expectation that an at-span, visually presented, verbal semantic processing task during the retention interval will disrupt the use of activated long-term memory for memory items, and it will have an impact on the use of phonological rehearsal because of the verbal content. Also the visual presentation of items for semantic processing will disrupt the supplementary use of any visual codes to support memory for the visually presented words. Therefore, dual-task recall performance will show a large reduction in performance relative to single-task memory. Processing performance will show a smaller reduction than memory for dual-task compared with single-task. For memory, there will be little interaction with age.^2^

There was the expectation that an at-span picture-based semantic processing task during the retention interval will disrupt the use of activated long-term memory, and any use of the passive visual store to support memory for the visually presented verbal items, but will have no impact on the use of phonological loop. Accordingly, it is expected that memory perfomance will be disrupted but to a lesser extent than with a verbal semantic processing tasks. So, there should be an interaction between dual task and type of semantic processing task on memory. Recall of the letter list will also show a smaller disruption under dual-task conditions for young participants than for the older participants. Any disruption of memory under dual-task conditions will primarily affect the memory for items, but have little effect on memory for the order of items that participants are able to recall. Processing performance will show little or no disruption under dual-task load for the younger group, but will show a small disruption for the older group. Finally, it was expected that both types of spatial processing task will have a small effect on recall performance relative to single task for both age groups. Processing performance will show no drop in performance under dual task for the younger group, but will show a small disruption in the older group.

### Embedded-process model expectations

Other theorists have suggested that there is a core limit that holds across different domains, such as a limit in the capacity of the focus of attention (Cowan, 1988). According to the embedded processes model of working memory (Cowan, 1988, 2005, 2010, 2016, 2019), attentional resources act as a selective filter to activate task-relevant representations from long-term memory. Entry into working memory is assumed to occur when a subset of information in the long-term store takes on a temporarily heightened state of activation. A further subset of the activated information can be made particularly salient when it falls under the focus of attention which can cover only a small amount of information at any given time. In addition, the embedded processes model assumes a central attentional controller that provides domain-general processing capacity (Cowan, 1999). The controller comprises both a voluntary central executive system and an involuntary system for the automatic recruitment of attention. Its role is to supervise covert processes that serve to maintain information over time by reactivating decaying memory representations. Subvocal rehearsal may serve as one such reactivating mechanism. However, the model additionally suggests that searching through a set of memory items by recurrently subjecting them to the focus of attention can also serve to refresh their representations (Cowan, 1999).

Cowan assumes that semantic processing requires retrieval from long-term memory, which should be carried out successfully, but at the expense of any concurrent task (based on findings of Craik et al., 1996; Rhodes et al., 2019), in this case, mnemonic processing to retain the letters in working memory. Spatial processing is presumed to require attention when rapid responding is required, but in a manner that allows participants to carry out spatial processing at a somewhat lower rate so as to continue to maintain letters in working memory to some degree; this expectation is in contrast to semantic retrieval, which presumably forces most of the dual-task cost to be absorbed by mnemonic maintenance. In this study, efforts have been made to reduce the low level visual similarity of the letters and words with the use of either lowercase or uppercase letters. As a result, a small amount of representational interference is predicted between the visually presented memoranda (letters) and the verbal processing items (words). In line with this, it is predicted that there will be a large dual-task cost for memory performance and little-to-no dual-task cost for the processing tasks requiring a semantic decision. The time spent making the “obligatory” semantic decision is time that cannot be spent maintaining the memory items. When the memory task is combined with spatial processing, in contrast, it is predicted that the cost for the memory task will be smaller and a reliable dual-task cost will be observed for processing. It is assumed that the spatial decision can be delayed to allow refreshing of the memoranda. This will lead to more errors/slower RTs for the processing task but allow better maintenance of the memoranda. Furthermore, it is predicted that dual-task costs in memory performance will be larger for verbal stimuli than visual, owing to interference, irrespective of whether the decision required is semantic or spatial.

For older adults the same general effects are predicted but are expected to be magnified due to deficits in switching between processing and maintenance (Wasylyshyn et al., 2011). The dual-task cost for memory with a semantic task will be amplified, as older adults are presumed to be slower to switch back to refreshing in-between processing items. Semantic tasks are presumed to be just as obligatory for older adults as young, so no disproportionate dual-task cost for semantic decisions is expected. For the spatial task, as discussed previously, there is a greater need for scheduling, as the processing decision can be delayed to allow for refreshing cycles. Thus, the switching cost experienced by older adults may be expected to apply to both tasks, leading to exacerbated dual-task costs, relative to the young, for both memory and processing. Furthermore, the spatial decision may become, to a degree, automatised for the younger adults over the course of the experiment and less so for the older adults (e.g., Hartley et al., 2011), which may increase the age differences in dual-task costs further. This theoretical view does not lead to any clear predictions of age differences with regard to representational interference, between the to-be-remembered letters and the processing task when words are used; phonological, semantic, and lexical representations are assumed to be activated in both cases.

### TBRS model expectations

The TBRS model (Barrouillet et al., 2004, 2007; Barrouillet & Camos, 2015) proposes that there is time-based sharing of attention between the storage of memory representations and the processing of incoming information. Like the embedded processes framework, this model presupposes that the maintenance of memory traces depends on their activation through attentional focusing. However, contrary to the embedded processes framework, the TBRS model assumes that a central bottleneck constrains cognitive activities that require attention, leading to a sequential functioning of working memory and the obligatory switching of attention between processing and maintenance activities. Within the TBRS model, recall performance on working memory tasks combining storage and processing depends on the interplay between intervals during which memory traces decay when attention is occupied by processing, and intervals during which attention is available for the maintenance of the memoranda. Crucially, the central (executive) attentional resource is assumed to be time-shared between processing and storage regardless of the nature and domain of the stimuli involved. That is, spatial storage is expected to be disrupted by both verbal and spatial processing activities and vice versa (e.g., Uittenhove et al., 2019; Vergauwe et al., 2010). Attentional refreshing, the maintenance mechanism that is interrupted by attention-demanding tasks, is described as separate from subvocal rehearsal (e.g., Camos, 2015, 2017, for review; Camos et al., 2009, 2017). Just as processing prevents attentional refreshing, leading to poorer recall performance, refreshing activities delay performance in processing tasks, with an increasing postponement of responses as memory load increases (e.g., Camos et al., 2019; Chen & Cowan, 2009; Vergauwe et al., 2014). This effect occurs only when the phonological loop is unavailable or when its capacity is exceeded.

In the TBRS model, the nature of the stimuli involved in the secondary task has little-to-no effect on the maintenance of the memory items. The only exception is when the memory items and the processing items have the same basic attributes (e.g., both letters or digits). In a similar vein, the nature of the secondary task has no effect on memory performance, its impact on the maintenance depends entirely on its cognitive load,^3^ irrespective of the processes involved (e.g., Barrouillet et al., 2007, 2011). Therefore, since the processing items differ from the memoranda, varying stimulus domain is expected to have no effect on memory performance. Moreover, despite the fact that the secondary task varies in terms of the requirement to access representations stored in long-term memory, such experimental manipulation is also assumed to have no effect on memory performance, because the titration procedure should lead to equalisation of the cognitive load between the secondary tasks. Hence, the only effect predicted by the TBRS model in memory performance is a dual-task cost: the requirement to perform a secondary task while maintaining memory items should lead to poorer recall performance. As the maintenance and the processing rely on the same attentional resource, the same predictions are made for the performance in the processing tasks. The titration procedure is likewise expected to equalise cognitive load across age groups, eliminating any age effects. In consequence, only a dual-task cost is expected. See Table 1 for a summary of all predictions.

**Table 1.** A summary of predictions for the current experiment put forth by the proponents of three working memory frameworks.

Effect and its ramifications	Multiple components	Embedded processes	TBRS
Task (single vs. dual) × Decision (spatial vs. semantic) *No interaction*			
Initial expectation:	For memory: larger dual-task cost for semantic than spatial judgements. For processing: larger dual-task cost for semantic than spatial judgements.	For memory: larger dual-task cost for semantic than spatial judgements. For processing: larger dual-task cost for spatial than semantic judgements.	No interaction
Principle behind expectation:	Semantic processing was assumed to require access to well-learned information in long-term memory and involve verbal processing of the stimulus. This will disrupt verbal memory more than a spatial task, which involves a different stimulus domain.	It was assumed that spatial but not semantic processing can be delayed to allow refreshing of the memoranda.	The impact of the processing task on memory performance was assumed to depend solely on its cognitive load, regardless of the processes involved. Titration procedure was expected to equalise cognitive load across task conditions.
Necessary modifications:	Spatial decisions may involve some verbal processing that was not expected. There may also be unintentional subvocalising of the word or picture name even if this is not required for the spatial task. Both could disrupt verbal memory more than anticipated if it was a purely spatial task such as visuo-motor tracking.	In retrospect, given the titration procedures and resulting time pressure, it may have been incorrect to believe that a spatial judgement could be delayed.	No change necessary.
Task (single vs. dual) × Stimulus (verbal vs. non-verbal) *For memory: no interaction. For processing: larger dual-task cost for verbal material.*			
Initial expectation:	For memory: larger dual-task cost when verbal material is being processed. For processing: Small dual-task costs for verbal but not for non-verbal.	For memory: larger dual-task cost when verbal material is being processed.	No interaction
Principle behind expectation:	It was assumed that interference will be domain-specific, so verbal processing will interfere with verbal memory and vice versa, but non-verbal processing will not show mutual interference with verbal memory.	A small amount of representational interference was predicted between the visually presented memoranda (letters) and the verbal processing items (words).	Processing items differed from the memoranda. Therefore, varying stimulus domain was expected to have no effect on memory performance.
Necessary modifications:	Spatial decisions for non-verbal material may involve some verbal processing that was not expected (e.g., subvocally saying “above/below” as a keypress is generated). There may also be unintentional subvocalising of the word or picture name even if this is not required for the task. Both could disrupt verbal memory. Holding verbal items in memory disrupts the verbal aspects of semantic processing and there is verbal processing of words even when not required to make spatial judgements.	It is not yet clear when dual-task effects fall on memory as in our previous findings and hence as predicted, versus on processing as in the case of this effect. It seems premature to offer an explanation of this discrepancy.	The semantic judgement task should require reading the items and hence may have impaired the verbal-specific maintenance mechanism.
Task (single vs. dual) × Age × Decision (spatial vs. semantic) *No interaction*			
Initial expectation:	No interaction	For memory: larger age effects when semantic decision is required. For processing: larger age effects for spatial than semantic judgements.	No interaction
Principle behind expectation:	Verbal abilities and access to semantic knowledge tend not to decline with age, so no age difference in dual-task cost is expected for combining verbal memory with semantic judgements. The combination of different domains for verbal memory and spatial judgements should compensate for any age-related decline in spatial abilities.	Older adults were presumed to be slower to switch back to refreshing in-between processing items. The switching cost experienced by older adults was expected to apply to both tasks.	Titration procedure was expected to equalise cognitive load across age groups, eliminating any age effects.
Necessary modifications:	No change necessary.	Given the absence of the expected task × decision interaction, no further change is needed to explain the absence of an age difference in this interaction.	No change necessary.
Task (single vs. dual) × Age × Stimulus (verbal vs. non-verbal) *For memory: larger dual-task cost in the verbal condition for younger (but not older) adults.*			
Initial expectation:	Older participants should show less impact of dual task for verbal semantic judgements than younger participants.	No interaction	No interaction
Principle behind expectation:	The enhancement of verbal and semantic knowledge from life experience in older adults.	No clear predictions of age differences with regard to representational interference.	Titration procedure was expected to equalise cognitive load across age groups, eliminating any age effects.
Necessary modifications:	No change necessary.	The effect seems largely due to differences in the baseline condition so further research is needed to clarify the effect.	No account for the age-related difference. Further studies are needed on the evolution across adulthood of the balance between the two maintenance mechanisms.
Task (single vs. dual) × Age × Decision (spatial vs. semantic) × Stimulus (verbal vs. non-verbal) *No interaction*			
Initial expectation:	For memory: age effect only for semantic judgements performed on non-verbal material, but not on verbal material. For processing: no age effect for semantic judgements performed on verbal material, but there will be a small age effect for semantic judgements with non-verbal material.	No interaction	No interaction
Principle behind expectation:	Verbal memory and processing do not decline with age, but non-verbal abilities do show age-related decline.	No clear predictions of age differences with regard to representational interference.	Titration procedure was expected to equalise cognitive load across age groups and task conditions, eliminating any age effects.
Necessary modifications:	Older people may compensate for any decline in non-verbal abilities by using verbal strategies with non-verbal material.	No change necessary.	No change necessary.

TBRS: time-based resource sharing.

A summary of the results of the experiment is given in the far left column and highlighted in italics. The table also contains the theoretical principles behind each expectation and explains how each theory has to change to accommodate the data. See main text for description of the other variables.

## Working towards a consensus on the mechanisms of working memory

Although, seemingly, a clear theoretical divide exists between working memory frameworks that see storage and processing as relatively independent, and those arguing for a common resource supporting both functions, the picture is more nuanced. The embedded processes and TBRS accounts clearly predict that storage and processing should compete for cognitive resources and that superior performance of one task should come at the expense of performance on the other. However, within the multiple-components approach, if the capacity of verbal storage is reached, additional items can be saved by transforming the information into visuospatial or semantic representations, at the expense of visuospatial or semantic aspects of processing. Thus, all three of the views presented hitherto predict interference between storage and processing under certain task conditions. In light of this convergence between approaches, a careful and systematic comparison of the models is needed. This, in turn, may stimulate the discussion necessary to develop an integrated model of working memory (see Cowan et al., 2020; Logie et al., in press-a). One particular advantage of embedding this theoretical discourse in the context of cognitive ageing is that it prompts the researchers to adapt their theories and generate predictions in situations not previously considered. In the present case, each of the theories had to generate some new predictions for adult ageing. With this in mind, it is expected that, at least to some extent, the pattern of findings will run counter to hypotheses but that the process of seeing how far each theory can or cannot go will ultimately advance the field because it will require theorists to reconsider some of their core assumptions.

## Method

### Participants

A total of 121 participants took part in the study with data collected independently and in parallel at two laboratories, one in Edinburgh, UK, and one in Columbia, Missouri, USA. At the U.K. site, data from two participants were excluded from analysis.^4^ The final sample included 119 participants (71.43% female) with 59 younger (age range: 18–30) and 60 older (age range: 64–84) healthy participants, with 59 (29 younger/30 older) tested at the Edinburgh site and 60 (30 younger/30 older) at the site in Columbia. Basic participant characteristics for the final sample are presented in Table 2.

**Table 2.** Participant characteristics and MoCA scores (*M* and *SD* in parenthesis) split by testing site, age group, and condition (i.e., type of stimuli used in the processing tasks).

Site	Age group	Condition	*N*	*N* (female)	Age	MoCA	YoE
UK	Older	Non-verbal	15	12	70.93 (3.24)	25.93 (2.15)	15.00 (2.00)
		Verbal	15	8	71.33 (3.89)	27.13 (1.64)	15.47 (1.96)
	Younger	Non-verbal	13	12	21.69 (1.75)	28.46 (1.51)	14.92 (2.18)
		Verbal	16	10	22.19 (3.10)	28.81 (1.05)	14.69 (2.18)
US	Older	Non-verbal	15	10	72.60 (5.26)	26.53 (2.26)	16.47 (3.52)
		Verbal	15	11	73.13 (4.78)	26.93 (2.12)	16.43 (2.53)
	Younger	Non-verbal	15	11	21.00 (3.74)	27.07 (2.15)	15.33 (2.41)
		Verbal	15	11	21.27 (3.51)	28.27 (1.44)	14.47 (1.73)

MoCA: Montreal Cognitive Assessment score; YoE: years of education.

Our preregistered sample size of 120 was chosen to allow for counterbalancing of the order of memory and processing tasks across sessions. The analyses plan involved model comparison where an indeterminate Bayesian information criterion (BIC; Schwarz, 1978) difference is indicative of a lack of power.

Participants were tested individually in a single testing session which lasted approximately 90 min. At the U.K. site, participants were recruited from the student population of the University of Edinburgh, the Psychology Research volunteer panel, and the wider community of Edinburgh. At the U.S. site, participants were recruited from the student population of the University of Missouri-Columbia and from the wider local community through the Participant Pool of the Memory and Cognitive Aging laboratory. Participants received an honorarium in return for taking part in the study (GBP15 in Edinburgh and US$15 in Columbia).

All participants were fluent speakers of English, with no history of neurological damage, no problems with hearing, and normal or corrected-to-normal vision. The Montreal Cognitive Assessment (MoCA; Nasreddine et al., 2005), which is a measure of global cognitive functioning, was administered to all participants to ensure that all volunteers, particularly those above the age of 65, showed no evidence of cognitive dysfunction incommensurate with normal ageing. No participants were excluded from the final analysis due to poor performance on the MoCA. For detailed exclusion criteria, refer to the full protocol (https://osf.io/srm36/).

As shown in Table 2, our sample appears to be typical for studies such as these (e.g., Hansen et al., 2017). There is a general drop in scores on the MoCA with increasing age. Analyses of variance performed on years of education and MoCA scores revealed a significant difference between younger and older adults in terms of years of education (14.85 and 15.84, respectively, *F*(1, 111) = 5.23, *p* = .024). For MoCA, we found higher scores for participants in the verbal condition (27.79) relative to participants allocated to the non-verbal condition (26.97), *F*(1, 111) = 5.73, *p* = .018.

### Stimuli and apparatus

The final stimulus set comprised 74 unique names/images of animals and 74 names/images of manufactured objects. All words had an age of acquisition rating <14 years (Gilhooly & Logie, 1980). Names of animals and manufactured objects were matched in terms of average frequency in the British National Corpus (https://corpus.byu.edu/bnc/) and in the Corpus of Contemporary American English (https://corpus.byu.edu/coca/).

All stimuli were presented on a grey background (R = G = B = 128) via a 23 in. Lenovo ThinkVision T2324p monitor with a 60-Hz refresh rate. Memory items consisted of a pool of 18 letters excluding vowels and the letters “w,” “y,” and “z.” In the memory task, letters were capitalised and presented in the Lucida Console font with a height of 1.3° of visual angle at an approximate viewing distance of 60 cm. In the processing task, participants were shown either words or images describing/depicting animals (e.g., a cat) and manufactured objects (e.g., a book). Names of animals and objects were all lowercase and presented in the Lucida Console font with a height of 2° of visual angle at an approximate viewing distance of 60 cm. All greyscale drawings (300 × 300 pixels) depicting animals and manufactured objects (5° × 5° and their names) were sourced from the MultiPic databank (http://www.bcbl.eu/databases/multipic; Duñabeitia et al., 2018). The images were presented on a white background ( of visual angle). Participants responded to the processing task via a button box (www.blackboxtoolkit.com). The experimental procedure was programmed using PsychoPy (Peirce, 2007, 2009). The materials for this experiment can be found at https://osf.io/srm36/.

### Design and procedure

The testing session proceeded in two parts: (a) titration and (b) single- and dual-task blocks. Task domain (i.e., verbal or non-verbal) was randomly manipulated between subjects. Task requirements (i.e., semantic judgements or spatial judgements) were manipulated within participants. The experimenter remained in the room during the experiment.

**Figure 1.**
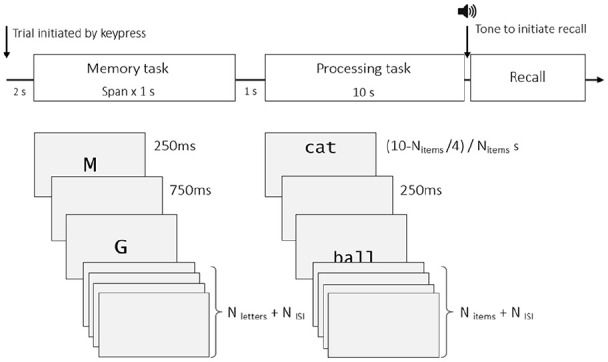
The general trial procedure. Only verbal task items presented (see Figure 2 for examples of processing stimuli).

The general trial procedures for memory and processing tasks are illustrated in Figure 1. Participants initiated each trial by pressing either of two keys on the response box, and this was followed by a 2,000 ms blank interval prior to the presentation of the first memory item. Each letter was presented at the centre of the screen for 250 ms followed by a 750 ms blank interval. This continued until the list of letters had been presented. Following the last letter there was a blank interval before the onset of the processing part of the trial. Following the processing interval a 400-Hz tone recall cue was played to prompt the participants to recall the letters in their correct serial order.

**Figure 2.**
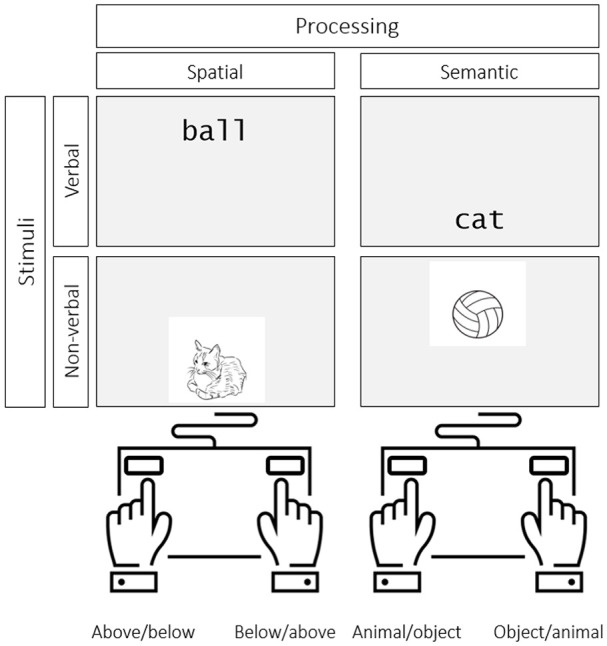
A matrix illustrating the four versions of the processing task based on the type of stimuli involved and the type of operation required. The key mapping was counterbalanced across participants.

Participants used the keyboard to enter responses. To acknowledge responses, each recalled item appeared on the screen for 500 ms, or until another key was pressed, at which point the most recently recalled item appeared in its place. Participants were informed that they could not correct mistakes and if they were unsure about a particular item, given the importance of recalling letters in serial order, they could type “0” (zero) to skip it. For the processing phase of the experiment, participants were required to complete two different activities, presented in separate blocks of trials (see Figure 2). Specifically, participants were instructed to make semantic animacy judgements (*Is it alive or manufactured?*) or (in separate blocks) decisions about spatial location of items presented on the screen (*Is it located above or below the centre of the screen?*) for a series of either verbal or non-verbal stimuli as quickly and as accurately as possible. Participants responded by pressing one of two keys on the button box to indicate their response. Depending on the number of processing items to be presented during the 10-s processing phase, each item appeared on the screen for (10–N/4)/N s, where N is number of items to judge, with a 250 ms blank interval in-between items. Participants were able to respond to a given item from its onset right up until the onset of the next word/image.

#### Titration of task difficulty

The purpose of the titration phase was to obtain a measure of each individual’s ability to perform the memory task and the processing tasks as single-tasks with placeholders presented in lieu of the omitted task. Participants completed a staircase procedure which began with either five letters to memorise or 12 words/images to categorise. Each level consisted of two trials with a given number of items. If participants were able to achieve 80% accuracy or greater across these two trials, they were deemed to have passed and an additional item was added for the next level. Otherwise, an item was taken away to reduce difficulty. This proceeded until the participant had completed at least eight levels (16 trials). If the eighth level was passed and it was the highest level passed by the participant, additional levels were run until the participant failed to reach the 80% accuracy criterion. The resulting span for the given task was the highest level passed by the participant during the titration procedure. At the beginning of each titration block participants had the opportunity to complete two practice trials (set at a list of five items for memory and at a list of 12 for processing). The order in which memory and processing tasks were titrated was counterbalanced across participants.

#### Single- and dual-task blocks

In the main part of the experiment the type of the cognitive operation required to complete the processing activities was manipulated. The order in which tasks requiring and not requiring access to long-term memory representations was counterbalanced across participants. To obtain a measure of performance on these tasks at span levels determined during the titration phase, single-task measures preceded dual-task blocks. In these single-task blocks, placeholders (i.e., black geometric shapes) were presented in place of the omitted task. Participants completed eight trials in each of the five main experimental blocks (comprising three single-task blocks and two dual-task blocks). Prior to each block participants were also given two practice trials.

Feedback on performance was presented following each trial in the form of animated bar plots that filled up to indicate the number of points, out of the 100 available (e.g., 50 for wholly accurate memory performance and 50 for wholly accurate processing performance in the dual-task blocks), that the participant obtained for each task. A running total of points out of the number possible to obtain in that block was also presented at the bottom of the screen. This approach to motivating participants has been effectively applied in several previous studies (Morey et al., 2011; Rhodes et al., 2019; Salthouse et al., 1984; Somberg & Salthouse, 1982). Participants were informed that to gain the most points they would have to be 100% accurate on both tasks.

### Statistical analyses

Data were analysed using generalised linear mixed-effects models (Bolker et al., 2009) with the lme4 package in R (Bates et al., 2015; R Core Team, 2018).^5^ Each model contained a random participant intercept to reflect differences in overall accuracy. We used the logit link function to model the log odds of a correct response on a given task. This scale is more appropriate when modelling accuracy, as it is bounded between 0 and 1, and it accounts for the fact that proportions are inherently more variable in the mid-range of accuracy (i.e., around 0.5). The function of the logit link is to take a linear combination of the covariate values and convert those values to the scale of a probability, that is, between 0 and 1. Therefore, it should be noted that while we plotted data on its observed scale, the modelling is done on log odds, a more appropriate latent scale (see Dixon, 2008, for further details on the appropriateness of this scale).

Note that when it comes to memory performance, the design was not fully factorial (i.e., single-task memory did not vary in terms of stimulus domain in the same way that the dual-task blocks did). Thus, in the main analysis of memory performance we grouped all memory conditions under one variable, *task*, and used orthogonal contrasts to test specific predictions. With this coding scheme, the first contrast compared single- and dual-task performance and the following contrasts compared within dual-task conditions (i.e., spatial and animacy judgements, verbal and non-verbal stimuli, and the variation in dual-task memory performance via the specific combination of stimulus and judgement). The analysis also included the effects-coded factor of age group (younger = −1, older = +1). In applying the model simplification procedure that we have used in previous experiments (Doherty et al., 2019; Rhodes et al., 2019), each contrast in the task variable was treated as a separate candidate to be removed. In addition, we conducted separate analyses of single- and dual-task data. For the single-task analysis, the test was of a main effect of age. For the dual-task memory accuracy data, the additional analysis included the variables of decision (spatial, semantic), stimuli (verbal, non-verbal), age (younger, older), and whether the task was dual or single, each effects coded. These analyses served as a check of the efficacy of the titration procedure in matching single-task accuracy across age groups and a further verification of any trends found in the more elaborately coded analyses included in the supplementary materials. For the analysis of processing performance, we had the variables of task (single, dual), decision (spatial, semantic), type of stimuli (verbal, non-verbal), and age group (younger, older) variable. Each of these factors was effects coded (level 1 of factor = −1, level 2 = +1).

Full models consisting of all main effects and interactions were simplified as follows. The highest order interaction was removed and the resulting model was compared with the full model via the BIC (Schwarz, 1978), which penalises the fit of a model for the number of parameters it has. If the BIC was lower for the reduced model this was considered evidence against the removed effect, in which case it was taken out for subsequent stages in the model comparison. This continued in a similar fashion through to the two-way interactions and then main effects. We did not consider removing interactions or main effects (e.g., A × B) if they were subsumed by retained higher order interactions (e.g., A × B × C).

Effects were scaled via the random participant effect standard deviation that was estimated along with the fixed effects. Thus, the effect sizes reflect the size of the effect relative to expected difference between individuals on the log odds scale of analysis. To translate these to a common scale, we used conventional criteria to refer to effects on the scale of expected individual differences (Cohen, 1988). Consequently, 0.2 of the average difference between individuals represents a small effect, 0.5 a medium effect, and 0.8 a large effect. While initially arbitrary, this nomenclature appears to be reasonable for effects sizes in research on memory (Morris & Fritz, 2013).^6^

## Results

### Memory and processing spans in the titration phase

“Span” for each of the tasks was estimated using a modified staircase procedure to find a level (defined as the number of letters/words/images) at which the participant was accurate approximately 80% of the time or more. For the memory task, responses were scored using a strict serial recall criterion: an accurate response required that both the letter and its serial position were correct. Accuracy for the processing task was defined as providing the correct binary response (either above/below the centre of the screen or alive/manufactured) within the allowed response window.

**Figure 3.**
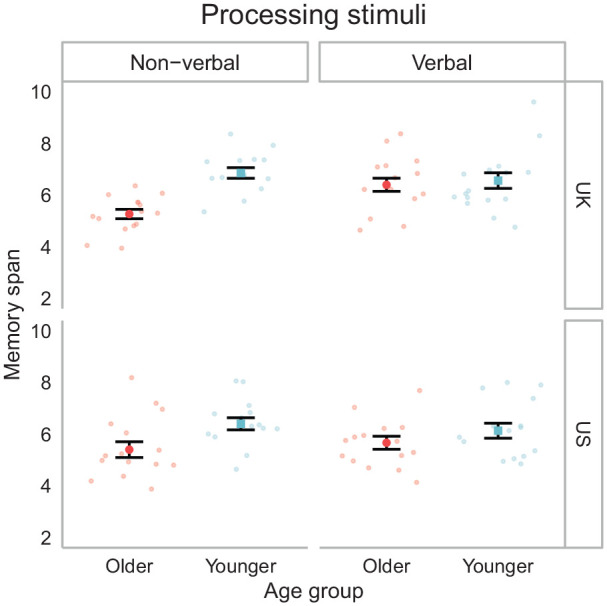
Memory spans by age group and processing stimuli during the titration phase. Note that the memory task (i.e., serial verbal recall of consonants) did not differ by processing stimuli but verbal and non-verbal conditions were completed by different samples of participants. Error bars indicate the standard error of the mean (SEM).

Figure 3 presents titrated memory spans split by the stimulus type of the processing task (verbal, non-verbal), testing site (United Kingdom, United States), and age group. Separate analyses of variance (ANOVAs) were conducted on the spans for the memory, spatial processing, and semantic processing tasks with the factors of age group, site, and processing stimulus (verbal, non-verbal). For memory, there was a clear main effect of age (*F*(1, 111) = 18.39, *p* < .001) with higher spans in the younger group (*M* = 6.47, *SD* = 1.04) relative to older (*M* = 5.68, *SD* = 1.05). Unexpectedly, there was an age group by stimulus interaction (*F*(1, 111) = 6.96, *p* = .010). As shown in Figure 3, this was due to a larger age difference with non-verbal relative to verbal stimuli. This was true for both the U.S. and the U.K. samples and is difficult to interpret as the memory task used for measuring single span task did not differ based on the group to which participants were allocated for the subsequent processing conditions. Speculatively, differences in initial memory performance may be attributed to the sampling differences for the between-participants nature of the comparison.

Turning now to processing performance, ANOVAs of spatial and semantic processing accuracy revealed clear effects of age (*F*(1, 111) = 53.96, *p* < .001, and *F*(1, 111) = 4.04, *p* = .047, respectively). Younger adults’ span in the spatial (*M* = 19.08, *SD* = 2.01) and semantic (*M* = 14.76, *SD* = 1.86) processing tasks was higher than that of older adults (spatial: *M* = 16.25, *SD* = 2.16; semantic: *M* = 14.08, *SD* = 1.80).

**Figure 4.**
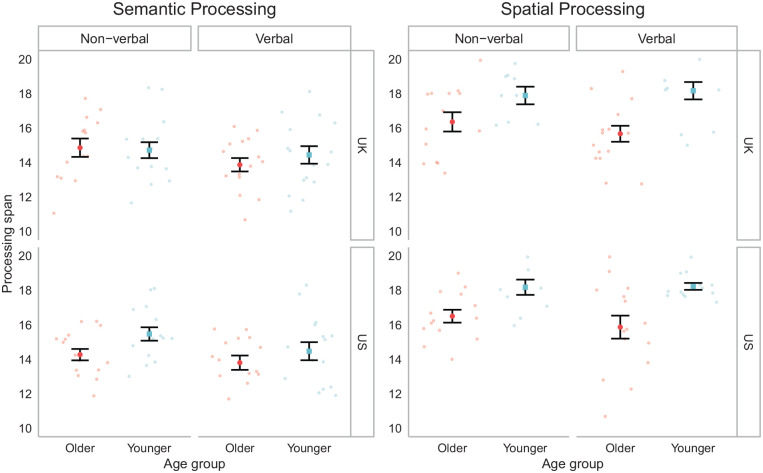
Processing spans by age group and processing stimuli during the titration phase. Error bars represent SEM.

As shown in Figure 4, age differences in processing span were more pronounced for the spatial task than for the semantic task. This pattern is consistent with previous evidence noted earlier that verbal abilities decline much less with age than do non-verbal abilities (Johnson et al., 2010; Park et al., 2002; Verhaeghen, 2003).

### Analysis of memory accuracy in the test phase

**Figure 5.**
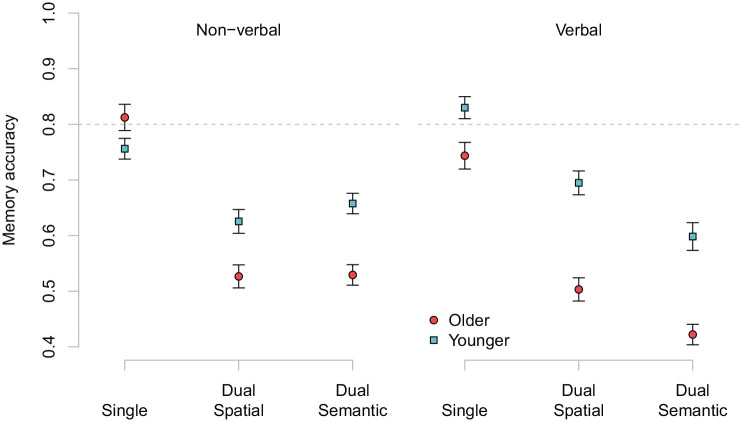
Memory accuracy for single- and dual-tasks by age and processing stimuli. Error bars represent standard error of the mean (SEM).

Memory accuracy data are presented in Figure 5. As described, previously, we constructed four contrasts: C1 single-task versus dual-task, C2 spatial judgements versus semantic judgements, C3 verbal stimuli versus non-verbal stimuli, and C2 × C3 interaction.

Table 3 summarises the results of the best-fitting statistical model in the analysis of memory accuracy (the step-by-step model selection procedure is described in the supplementary materials). There were clear main effects of single-task versus dual-task and spatial versus semantic judgements on memory accuracy, and a smaller effect of stimuli. Furthermore, the interaction term (C2 × C3) was significant, pointing, as shown in Figure 5, to poorer memory performance when combined with semantic judgements about verbal stimuli than spatial judgements about verbal stimuli. This interaction did not appear when non-verbal processing stimuli were presented, with the same dual-task cost to memory from spatial judgements as from semantic judgements. Crucially, there was a significant interaction between age and the single- versus dual-task contrast, indicating larger concurrence cost with advancing age. There was also an interaction between age and the third contrast (verbal stimuli vs. non-verbal stimuli), suggesting that for older adults, overall dual-task memory performance was poorer when combined with verbal processing material than when combined with non-verbal processing material, whereas there was no difference for younger adults. Age did not significantly interact with the decision (semantic or spatial) by stimulus (verbal or non-verbal) interaction contrast, providing no support for the idea that age differences in dual-task costs for memory performance were exacerbated by a particular combination of type of processing material and type of decision to be made for processing.

**Table 3.** Final best-fitting model for the analysis of memory accuracy.

Parameter	β	*SE*	z	*p*
(Intercept)	0.536	0.056	9.488	.000
C1 single versus dual	−0.831	0.031	−27.128	.000
C2 spatial versus semantic	−0.159	0.039	−4.031	.000
C3 verbal versus non-verbal	0.149	0.068	2.169	.030
C2 × C3	0.475	0.079	6.024	.000
Age group	−0.279	0.056	−4.950	.000
Age Group × C1	−0.200	0.031	−6.551	.000
Age Group × C2	−0.001	0.039	−0.037	.970
Age Group × C3	−0.174	0.070	−2.491	.013
Age Group × C2 × C3	−0.081	0.079	−1.031	.303

C components refer to specific contrasts between conditions. The first contrast compared single- and dual-task performance and the following contrasts compared within dual-task conditions. See the main text for description of the analysis approach.

Note that Figure 5 depicts baseline (single-task) differences between younger and older adults, particularly for participants randomly allocated to the verbal processing stimulus condition. This suggests that the titration procedure may have been less successful for younger adults in the verbal condition. However, this was not accounted for in the previous analysis, as stimulus type was only coded for the dual-task conditions. Thus, an additional analysis was conducted in which stimulus was included to code whether a particular participant was assigned to the verbal or non-verbal condition (coded: *non-verbal* = −1, *verbal* = 1). In addition there were the factors of whether the task was dual or single (coded: *single* = 1, *dual* = −1), age group (coded: *younger* = −1, *older* = 1), and site (coded: *UK* = 1, *US* = −1). This analysis, therefore, can tell us whether there were differences in dual-task costs between groups of participants randomly allocated to the verbal or non-verbal stimulus conditions (even though this factor did not influence the memory single task). Table 4 presents the final model from this analysis (see supplementary materials for additional analyses, including an analysis of dual-task costs that shows a main effect of age group).

**Table 4.** Final best-fitting model for the alternative analysis of memory accuracy.

Parameter	β	*SE*	z	*p*
(Intercept)	0.844	0.055	15.478	.000
Stimulus (verbal vs. non-verbal)	−0.023	0.054	−0.413	.679
Task (single vs. dual)	0.523	0.019	27.219	.000
Age group	−0.197	0.054	−3.622	.000
Stimulus × Task	0.039	0.019	2.041	.041
Stimulus × Age Group	−0.151	0.054	−2.778	.005
Task × Age Group	0.125	0.019	6.520	.000
Stimulus × Task × Age Group	−0.075	0.019	−3.917	.000

See the main text for description of the analysis approach.

Of note is the stimulus by task by age interaction pointing to a larger overall age difference in the verbal condition. Looking at the average memory accuracy, dual-task cost (i.e., single–dual) was more or less equivalent for older adults across the two stimulus conditions (verbal: 0.281, non-verbal: 0.284). For younger adults, the concurrence cost was larger in the verbal condition (0.183) than in the non-verbal condition (0.114), hence the smaller age difference. That is, the three-way interaction suggests that the age difference in the concurrence cost was somewhat smaller in the verbal condition. These results differ from those presented in Table 3, as now we are taking the difference in single-task baselines into account (see Figure 5 average memory accuracy). We also attempted to control for baseline differences statistically by using proportional change scores ([single − dual]/single), as has been used in some previous studies on age comparisons for dual-task performance (e.g., Logie et al., 2004). In line with the main analysis, an ANOVA on these scores revealed significant main effects of age group and decision (spatial vs. semantic), and a decision by stimulus interaction. Detailed results for the analysis of proportional changes scores are reported in the supplement.

### Analysis of processing accuracy in the test phase

**Figure 6.**
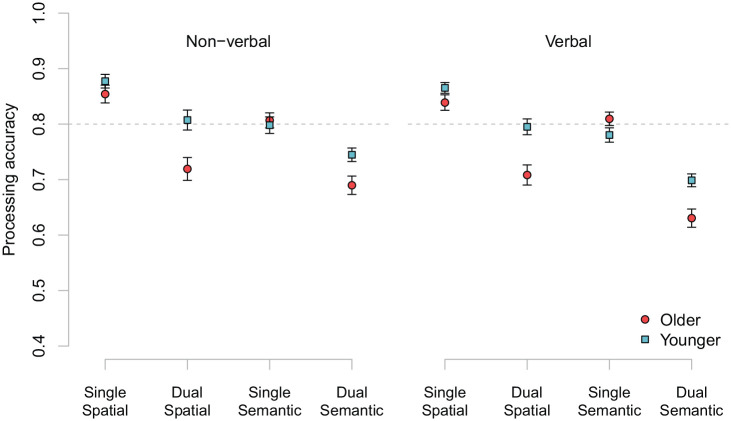
Processing accuracy for single and dual tasks by age and processing stimuli. Error bars are SEM.

Processing accuracy across experimental conditions and groups is shown in Figure 6. The analysis of processing performance included factors of age group (coded: *younger* = −1, *older* = 1), site (*UK* = 1, *US* = −1), processing stimulus type (coded: *verbal* = 1, *non-verbal* = −1), decision type (coded: *semantic* = −1, *spatial* = 1), and task (coded: *single* = 1, *dual* = −1). Table 5 presents the results of the best-performing model in the analysis of processing accuracy (the full model and description of steps taken in simplifying it can be found in the supplement). There was a clear main effect of decision type, with poorer performance for semantic than for spatial judgements. Note that titration procedure may have been less effective for the spatial task, where participants performed well above the 80% accuracy criterion in the single-task condition (see Figure 6). Main effects of task, reflecting the cost to processing of holding a verbal sequence in memory, and age group, with poorer performance for the older participants, were also significant.

**Table 5.** Final best-fitting model for the analysis of processing accuracy.

Parameter	β	*SE*	z	*p*
(Intercept)	1.311	0.033	39.386	.000
Decision (spatial vs. semantic)	0.202	0.010	19.779	.000
Task (single vs. dual)	0.307	0.010	30.203	.000
Stimulus (verbal vs. non-verbal)	−0.059	0.033	−1.784	.074
Age group	−0.098	0.033	−2.938	.003
Site (UK vs. US)	−0.006	0.033	−0.194	.846
Decision × Task	0.019	0.010	1.909	.056
Decision × Stimulus	0.017	0.010	1.700	.089
Task × Stimulus	0.025	0.010	2.444	.015
Decision × Age Group	−0.057	0.010	−5.631	.000
Task × Age Group	0.083	0.010	8.129	.000
Decision × Site	0.019	0.010	1.932	.053
Stimulus × Site	0.020	0.033	0.592	.554
Age Group × Site	0.013	0.033	0.401	.688
Decision × Task × Stimulus	−0.031	0.010	−3.040	.002
Decision × Stimulus × Site	0.069	0.010	6.814	.000
Decision × Age Group × Site	0.036	0.010	3.606	.000

See the main text for description of the analysis approach.

Turning to interactions, there was a decision by age group interaction effect with, overall, a larger age-related difference in performance of spatial, rather than semantic, judgements. There was a clear age by task interaction pointing to a larger dual-task cost to overall processing accuracy for older compared with the younger adults. There was also a three-way interaction between task, decision type, and stimulus domain. The dual-task cost did not vary greatly as a function of stimulus type when spatial decisions were required (single–dual equals 0.103 for non-verbal stimuli and 0.100 for verbal stimuli) but did for the semantic task, with larger dual-task cost for verbal (0.130) relative to non-verbal stimuli (0.087). Furthermore, there were two interactions including testing site. First, there was a three-way interaction between site, stimulus domain, and decision type. This was driven by a large difference in performance between the spatial and semantic tasks in the United Kingdom, specifically with verbal processing stimuli (0.102 compared with 0.041 in the United States). Second, there was a three-way interaction between decision type, age group, and testing site. This effect was largely due to a greater performance gap between younger and older participants in the difference between spatial and semantic judgements in the United States (0.055) relative to the United Kingdom (0.016). An ANOVA performed on the proportional change scores revealed only a significant main effect of age group (see supplementary materials).

### Scaled effect size for dual-task cost in accuracy

To compare the magnitude of the overall dual-task cost between the memory and processing tasks, we calculated scaled effect sizes by dividing coefficients by the estimated standard deviation (*SD*) of the participant random effect. Effect sizes for the overall dual-task cost and its interaction with age group are presented in Table 6. The effect sizes for the memory task are based on the follow-up analyses, which accounts for the different single-task performance between the participants assigned to verbal and non-verbal conditions.

**Table 6.** Select effect sizes and 95% confidence intervals for memory and processing task.

	Memory	Processing
Single versus dual	1.89 [1.75, 2.02]	1.78 [1.67, 1.90]
Younger versus older	0.71 [0.33, 1.10]	0.57 [0.19, 0.95]
Age-related increase in dual-task cost	0.45 [0.32, 0.59]	0.48 [0.36, 0.59]

*Note.* Effect sizes are based on scaling model coefficients and Wald CIs by the estimated *SD* of the participant random effect. This was 0.55 for the memory analysis and 0.34 for the processing analysis.

## Discussion

Disagreement is a forceful engine of scientific advance. It helps us define conceptual boundaries, draws our attention to neglected or contentious issues, and, crucially, prompts the design of pivotal experiments. However, the precise manner in which scientific disagreements are conducted can take many forms. Typically, the opposing sides state their respective positions and then throw rhetorical punches at one another. We chose a different approach. Reported here is a rare occurence of co-investigators who hold different views, working together on a collaboratively designed project to examine the extent to which memory and processing compete for cognitive resources and how this changes as people grow older. Specifically, we sought to assess whether age-related dual-task costs can be minimised or exacerbated by varying the nature of the processing activity in terms of stimulus domain and the need to access semantic representations stored in long-term memory. Our secondary aim was to clarify how storage and processing interact in working memory by evaluating our findings in the light of three competing theoretical frameworks.

The results reported here provide strong evidence that older adults’ performance suffers more than that of younger adults in dual tasks that require concurrent storage of letter sequences and semantic or spatial decisions. The key findings can be summarised as follows: Despite titrating single-task performance across age groups, clear dual-task costs were observed for both memory and processing performance. The data revealed a large age difference in the ability to store information over brief intervals, irrespective of the nature of the processing demands (i.e., concurrent spatial or semantic processing). This difference in memory performance between age groups was less marked when the processing tasks involved verbal material compared with non-verbal material. For processing performance, the interaction between age and the single- versus dual-task contrast was also significant, indicating a larger drop in processing performance for older than younger adults when processing was performed concurrently with retaining an at-span letter sequence. These age differences in dual-task processing performance did not vary as a function of the processing task domain (verbal, non-verbal) and requirements (spatial decisions, semantic decisions). The precise combination of results was not anticipated ahead of time. While each of the frameworks considered predicted some of the observed trends, no one set of predictions (see Table 1) completely matched all of the results obtained.

In the sections that follow, we evaluate our findings in relation to the three competing theoretical frameworks of working memory that motivated our study. We discuss how each of the theories could be modified in response to the data reported here. But first we consider our findings in the context of the wider literature on cognitive ageing and working memory and offer suggestions for future lines of enquiry.

### Age-related dual-task costs

A wide range of memory and processing tasks are used in the working memory literature, and they differ in the cognitive demands they place on the participant. We speculated that these different demands may systematically influence the extent to which older adults can successfully balance storage and processing of information. We found that a verbal processing task produced *smaller* age effects on memory performance than the corresponding task using non-verbal material. This is in line with previous findings showing that age effects in the verbal domain tend to be less marked than those observed in the visuospatial domain (e.g., Jaroslawska & Rhodes, 2019; Jenkins et al., 1999; Johnson et al., 2010; Myerson et al., 2003). Furthermore, we found no evidence to suggest that age differences in concurrent storage and processing performance were exacerbated when two tasks overlapped in domain (i.e., when both used verbal materials). Our results are thus consistent with previous studies suggesting that domain overlap may not inordinately affect older adults’ ability to simultaneously store and process (Jenkins et al., 2000).

As noted earlier, in a previous study, Rhodes et al. (2019) found an asymmetrical pattern of age-related deficits, which appeared strongly for the memory task but were not at all clear for the processing task. The authors conjectured that this skewed outcome may have been underpinned by the choice of processing activity. As mentioned in the Introduction, Rhodes et al. (2019) instructed participants to categorise outcomes of simple additions (e.g., 9 + 4 = 12?) as either correct or incorrect. It was argued that such retrieval of simple sums is largely automatic, and thus the dual-task cost was seen exclusively for the storage task. Here we tested this interpretation using processing tasks that required either purely spatial judgements about the location of the stimulus on the screen or were contingent upon access to semantic information stored in long-term memory. Whereas Rhodes et al. (2019) found a dual-task cost primarily on memory, the present manipulation resulted in clear dual-task costs on processing performance as well as memory. Moreover, there was no evidence to suggest that age differences in dual-task performance were attenuated when simple semantic (rather than spatial) decisions were required. Given that the demands of each single task were titrated to the ability of each participant, this suggests that cost is specifically the result of having to perform two tasks concurrently in our paradigm, not because the groups differed in their single-task performance, or because of any differential cognitive demand between the different processing tasks. The greater dual-task costs in older adults require further investigation before we will understand the role of processing capacities, speeds, and strategies in producing these results.

Contrary to expectations, age differences in dual-task performance were not diminished when participants had to make semantic judgements compared with spatial judgements. A possible explanation for this result is that the categorisation of verbal stimuli as either living or manufactured might place a greater demand on cognitive resources (regardless of our preferred theory) than we had originally assumed. This increased difficulty of the semantic task when verbal items (but not non-verbal items) are presented may be due to the fact that words have to be read prior to making a semantic decision—a requirement that is not necessary when making judgements about spatial locations. This reading requirement might in turn affect rehearsal which is involved in the concurrent maintenance of the letters. Moreover, although the objects and animals were very familiar to all participants, it may be that living/non living is only one of many properties of each item stored in long-term memory, and so retrieval of the requested property is not without some cognitive cost (e.g., Collins & Quillian, 1969).

Our findings are at odds with recent meta-analytic data, suggesting that titration of task difficulty mitigates age differences in dual-task costs (Jaroslawska & Rhodes, 2019). However, the pattern of findings reported here and in recent papers (Bier et al., 2017; Rhodes et al., 2019) seems to suggest that other factors in addition to the lack of titration result in age-related dual-task effects, and factors other than domain overlap between processing and storage material to be combined could be crucial. For example, in many of the studies reporting a lack of an age-related dual-task cost in either memory or processing, the titrated verbal memory load was combined with a titrated perceptuo-motor tracking task (e.g., Baddeley et al., 1986; Logie et al., 2004). This apparent inconsistency highlights the need for future studies to develop standardised procedures for estimating each individual’s ability to perform the concurrent tasks in isolation, and for identifying which combinations of tasks result in age-related dual-task costs, and which do not. Directly comparing age differences in working memory dual-task costs under titrated and non-titrated conditions might offer another potential avenue for future research. Another possible target for future research may involve replicating the current findings using a fully crossed design including both verbal and non-verbal processing items *and* verbal and non-verbal memoranda. However, as noted by Logie (2018), there is no guarantee that participants will perform tasks as expected by the experimenter. For example, tasks that are assumed to involve spatial memory or spatial processing may be performed by participants using verbal memory and verbal processing, and this would undermine any attempt to investigate cross-domain versus within-domain memory and processing.

Before discussing the implications of the findings, it is important to consider the potential methodological limitations associated with the use of extreme-groups design. Although sampling participants from the extremes of the distribution is very common in the area of cognitive ageing, methodologists have noted several limitations associated with this method, including artificially inflated effect size and compromised reliability (e.g., Preacher, 2014). Our choice of method was motivated, in part, by a previous study conducted by Rhodes et al. (2019), where age was a continuous variable and the dual-task age effects appeared to be linear across age as a continuous, and not a grouping variable. This implies that Rhodes et al. (2019) would have come to the same conclusions using an extreme groups design. Nevertheless, future studies could be improved by the inclusion of a lifespan sample.

### Resource-independence and resource-sharing in working memory

A key strength of this experiment is the inclusion of a set of predictions based on three competing theoretical frameworks for working memory. As the precise combination of results was not anticipated by any of the three theoretical frameworks, we next discuss the predictions and where each framework hit and missed the mark. To recap, only the TBRS model correctly predicted that dual-task costs would be equivalent, irrespective of the type of judgements required during processing. This prediction was based on the assumption that the precise nature of the processing activity does not affect memory performance. Rather, its impact on the maintenance activities depends solely on its cognitive load, regardless of the processes involved. It was further assumed by the TBRS model that the titration procedure would equalise the cognitive load imposed by the memory and processing activities across age groups, predicting no age-related differences, and contrary to this model, clear age-related dual-task costs were found.

Counter to hypotheses put forward by both multiple-component and embedded process accounts, we found no evidence of a larger overall dual-task costs to memory performance when processing tasks used verbal materials. This lack of interaction was anticipated only by the TBRS model. None of the theories accurately predicted the interactions between dual-task costs and stimulus domain or larger dual-task costs for verbal materials for processing performance. With respect to age-related effects, none of the predictions fully accounted for a three-way interaction between dual-task costs, age, and stimulus domain. The unanticipated finding was that of a larger dual-task cost to memory performance in the verbal condition for younger, but not older adults. However, this is consistent with the multiple-components assumption that older adults might gain some advantage from the lack of age-related decline in verbal abilities and the enhancement of access to semantic memory from accumulated life experience. Note that this was driven by the semantic and not the spatial task. Furthermore, we found no evidence to suggest that the age-related deficit in dual-task performance was attenuated when the processing task involved the retrieval of information from long-term memory. This outcome was predicted by the TBRS and multiple-component frameworks. Finally, our data do not support the idea that age-related deficit in dual-tasking is exacerbated by a particular combination of stimulus type and judgement. This outcome matched the predictions put forward by the embedded processes and TBRS accounts.

Clearly, each theory requires some reconsideration of its core assumptions, or at least under what circumstances expected effects should be observed. The TBRS model anticipated the important finding that the dual-task costs incurred by the memory task did not differ as a function of the type of judgement to be performed, either spatial or semantic. This was based on the assumption that as long as both types of tasks involve the same cognitive load, that should result from the titration procedure, the TBRS model predicts the same detrimental effect on memory performance, as already observed in previous studies (Barrouillet et al., 2007, 2011). By contrast, unanticipated was the greater dual-task cost in older adults, whatever the task to be performed and the material presented. The erroneous prediction from the TBRS account followed the same rationale as the previous one, that is, the titration procedure should equate cognitive load across age groups. However, this did not consider the importance of one of the key mechanisms hypothesised by the TBRS account, which is the rapid switching process between processing and refreshing the items in memory. Because the TBRS model assumes that only one process can take place at a time due to a central bottleneck, attention must be rapidly switched from storage to processing and from processing to storage and during the short pauses that can be freed during processing (Barrouillet et al., 2004, 2011; Barrouillet & Camos, 2001, 2015). The greater dual-task costs observed in older adults might result from a slower switching process in this age group. Slowed processing speed is a hallmark of cognitive ageing (Salthouse, 1996), and the titration procedure could not compensate for a slower switching process because the titrated (single) tasks did not require a switch between processing and storage. Even at equivalent cognitive load, a slower switching process would result in a sub-optimal use of the periods of free time available for refreshing decaying memory traces. This can then account for the greater dual-task costs incurred by older adults. The other finding that the TBRS authors did not anticipate is a larger dual-task cost to memory performance in the verbal condition for younger, but not older adults. Note that this is mainly due to the semantic task, and not to the spatial task (Figure 5). In both age groups, the dual-task cost was larger in the semantic task with verbal material than in any of the other conditions, and this increase was more pronounced for younger participants. This greater dual-task cost could have been anticipated by the TBRS model with a more careful analysis of the designed tasks. A key feature of a semantic task with words is the need to read the words for a semantic judgement, reading being unnecessary in the spatial task or for semantic judgements from images. Even if the titration procedure had equated the cognitive load of the four tasks, the necessary reading activity might have involved the articulatory loop assumed by the TBRS model to contribute, along with the executive loop, to the maintenance of verbal material. This could explain, within the TBRS approach, why performing semantic judgements on verbal stimuli had a more detrimental effect on verbal maintenance than the other tasks. However, it seems that there is nothing in the current version of the TBRS model that can explain why this difference was more pronounced in the younger participants. Maybe some exploration of the evolution with age of the balance between the two mechanisms of verbal maintenance (i.e., the executive and the articulatory loops) would be a fruitful avenue for future research.

The embedded-processes framework was successful in predicting dual-task costs generally. A core concept in this approach is the sharing of the focus of attention between storage and many kinds of processing. However, some of the auxiliary assumptions gleaned from recent studies did not correctly generalise to this study. In memory, it was expected that the similar use of words in the storage task and the processing task would result in inter-task featural interference with memory, regardless of the nature of the processing task. This prediction came true when the processing task was semantic, but not when the processing task was spatial. One altered assumption that could explain these results is that participants are able to filter out the verbal nature of stimuli in the processing task when the judgement that needs to be made is spatial rather than semantic. This was not expected inasmuch as, for example, participants cannot ignore the verbal nature of words in a Stroop task (Stroop, 1935). In that kind of task, though, perhaps more foveal gaze is needed to the word to name the colour, compared with the present spatial location task, and the absence of foveal gaze might allow filtering out of the verbal information. In processing, it was expected that semantic retrieval from long-term memory would automatically recruit attention, as it has appeared to do in other tasks (Ricker et al., 2010). Doherty et al. (2019) found that one kind of retrieval, presumably needed for arithmetic, resulted in dual-task effects on memory, but almost no effects on the arithmetic task itself. A comparable pattern was therefore predicted here for letter memory combined with semantic processing but, instead, the dual-task cost was distributed across both tasks, as it was in the spatial processing conditions. A revised assumption that could account for these findings is that arithmetic requires a longer-lasting duration of attention with each processing episode, leaving little opportunity for rapid refreshment or rehearsal of the memory materials, in contrast to the present semantic task. Decision about the category of a word or object might occur quickly enough that there is more of an opportunity to switch attention back and forth between semantic judgement and mnemonic processing of the letters, distributing the dual-task cost across memory and processing. In sum, the embedded-processes approach worked in its fundamental assumption about dual-task costs, but failed in other detailed assumptions on which the theory has been silent. The embedded-processes model was not consistently able to predict the magnitude of featural interference independent of the processing task, or to predict whether dual-task costs would fall upon memory or be distributed across memory and processing.

The multiple-component framework predicted that verbal processing would involve the phonological loop, and hence this would undermine rehearsal of the verbal memory items. As noted in the Introduction, this model assumes that verbal processing and verbal memory abilities are largely unaffected in healthy ageing, whereas executive functions and visuospatial function decline from early adulthood, and older people appear to use verbal strategies to perform visuospatial tasks (e.g., Forsberg et al., 2020; Johnson et al., 2010; Myerson et al., 2003). This could account for the smaller difference in older participants than in younger participants between the impact on memory of semantic judgements with verbal items compared with the other judgements required. The multiple-component framework did not anticipate the large age-related dual-task cost or the finding that semantic judgements were more disruptive than spatial judgements overall. It is possible to propose an account within multiple components by a post hoc analysis of the possible demands of each task and task combination. Predictions for empirical outcomes are based on assumptions about what cognitive functions might be required to perform a given task. If those assumptions are wrong, and participants perform tasks in ways that are not expected, then the predicted outcomes will not be obtained. However, the results that are observed can be used to generate hypotheses for testing in future experiments. The assumptions for the tasks for the current experiment were extrapolated from previous experiments in which a verbal memory load was combined with perceptuo-motor tracking (e.g., Baddeley et al., 1986; Logie et al., 2004), and from previous findings that a cognitive load does not appear to disrupt semantic retrieval (e.g., Baddeley et al., 1984). However, the processing tasks here were very different from those in previous studies, and left open the possibility that during the spatial task, participants subvocalised verbal labels for their actions (e.g., above–below). This could have acted like articulatory suppression that many previous studies have shown to disrupt serial-ordered verbal recall. In the perceptuo-motor tracking task used in previous studies, subvocalisation would have been much less likely. These differences from previous studies could be crucial.

In sum, it is clear that to progress further towards creating a well-performing theoretical framewrok for working memory, we will need a general framework that proves to be useful in accounting for a wide range of data, as well as in enhancing our understanding of working memory in everyday tasks. Future research involving this form of “adversarial collaboration” would aid the development of a more integrated model by developing more accurate indices of which components of a multiple-component system, or which aspects of attention, are taxed during particular experimental procedures.

## Conclusion

For both memory and processing performance, there is a large drop in performance between single and dual tasks, regardless of the experimental condition. This dual-task cost increases with age. With respect to moderators, stimulus domain appeared to play a role in modulating the extent of age difference in memory performance, with poorer recall performance when combined with non-verbal processing materials. Age did not interact with the decision (spatial, semantic) by stimulus (verbal, non-verbal) interaction, providing no evidence that age-related decrements to concurrent memory performance are amplified by a particular combination of processing materials and the mental operations required to complete the task. Another tangible outcome of the process of adverserial collaboration exemplified in this work is that it pushed theorists to revisit their key theoretical assumptions and to specify the features of storage and processing tasks used in dual-task paradigms in a more precise way.

So where shall we go from here? Given that each of the theoretical frameworks of working memory predicted some of the observed effects, but no account predicted the complete pattern of results, one way forward may be to integrate the models to handle the unexpected effects (e.g., the absence of some of the expected material-specific effects). This modus operandi, however, may not be the most parsimonious solution. An alternative course of action may be to, at least temporarily, brush aside the details of existing theoretical frameworks and focus instead on the domains of several key principles that are fundamental within the theories. As a starting point, this proposed “meta-theory” of working memory should probe (a) material-specific limits in interference, capacity, or time, (b) domain-specific rehearsal mechanisms, (c) a general attention-related capacity limit, (d) a general attention-related refreshing speed limit, and (e) the contribution of long-term memory. This approach could pave the way towards a single adequate and elegant solution, with the proviso that the next step is then to determine the boundaries of each domain, and any common ground that can be shared or traded off between them.

## Notes

1. Preregistered predictions are available on the Open Science Framework (https://osf.io/srm36/).

2. An additional multiple components prediction registered in advance was that any disruption of memory under dual-task conditions will primarily affect memory for items but have little effect on memory for the order of items that participants are able to recall. This was based on findings from Saito et al. (2008) and Logie et al. (2016), and on a measure of order, independent of item recall devised by Nimmo and Roodenrys (2004). However, this analyses is outside the scope of this article which is focused on age-related dual-task costs, so will be addressed elsewhere.

3. The only exception is when the secondary task is performed out loud, which impairs the supplementary maintenance through subvocal rehearsal.

4. One data set was incomplete as a result of technical difficulties. Data from another participant were excluded due to their familiarity with the Montreal Cognitive Assessment (MoCA; Nasreddine et al., 2005) used as a screening test for possible cognitive impairment.

5. This article was written using the R package papaja (Aust & Barth, 2018).

6. This analysis plan was specified before we began data collection. More detail is available at https://osf.io/srm36/.
